# Subconjunctival Emphysema After Descemet's Stripping Automated Endothelial Keratoplasty (DSAEK) 

**DOI:** 10.2174/1874364100802010107

**Published:** 2008-05-23

**Authors:** Takeshi Ide, George D Kymionis, Sonia H Yoo, Terrence P O’Brien

**Affiliations:** Bascom Palmer Eye Institute, University of Miami, Miami, FL, USA

## Abstract

DSAEK has recently evolved as a preferred method in place of PKP. After DSAEK, we experienced subconjunctival emphysema, decreased air bubble in the AC and low IOP. This could lead to complications, higher rate of dislocation or infection. We should control the wound closure with suture when in doubt.

## INTRODUCTION

Penetrating keratoplasty (PKP) has long been the procedure of first choice for replacement of diseased endothelium. In recent years, however, attempts have been made to replace only the endothelial layer [1,2]. Descemet's Stripping Automated Endothelial Keratoplasty (DSAEK) has recently been popularized [3-5]. Fewer complications are expected when posterior corneal tissue is selectively replaced, because full thickness transplantation can be complicated by a variety of postoperative problems.

In place of running or interrupted sutures in PKP, in DSAEK we inject air into the anterior chamber to push the donor cornea against the host stroma. Air is known to cause damage on corneal endothelium [6,7] and, therefore, some doctors aspirate air several minutes after air injection. Most doctors, however, leave the air in the anterior chamber (AC) as long as possible, in case DSAEK donor button dislocates in the AC. Air maintenance in the anterior chamber is critical for this surgery within the first few hours, especially after re-bubbling.

In this case report, we observed sub conjunctival air bubbles and reduced AC air bubbles on post-operative day 1.

## CASE REPORT

An 85 year-old lady with Fuch’s dystrophy had DSAEK surgery on her left eye at our center. She had previous cataract surgery and her intraocular lens was centered in her posterior capsule. Her ophthalmologic and systemic general condition was otherwise normal.

The DSAEK surgery was routine and uneventful. At the end of DSAEK surgery, we injected air into the anterior chamber.

**Fig. (1):**
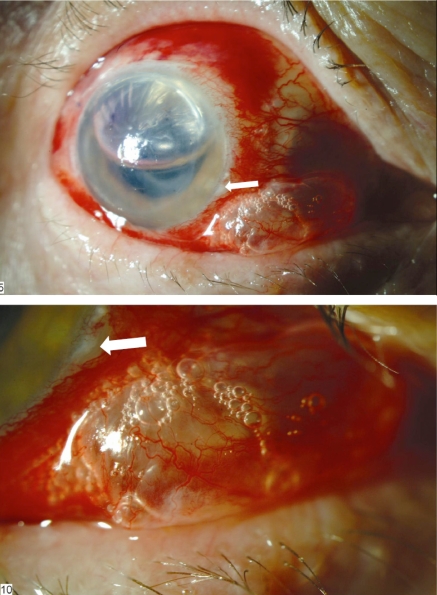
Subconjunctival emphysema after DSAEK. Subconjunctival air bubbles and reduced air bubbles in AC are seen in the left eye. And paracentesis incision (arrow) can be seen over the subconjunctival sclera.

The next day, slit lamp examination showed subconjunctival emphysema in the temporal-inferior part of the left eye. The DSAEK tissue in the anterior chamber had wrinkles but adhered well. The anterior chamber was about 50% filled with air. This reduction was faster than usual. The intraocular pressure (IOP) was 14mmHg and 7mmHg in her right and left eye respectively. The ocular findings were otherwise normal. The entry of paracentesis incision in the temporal-inferior part could be seen just over the sclera-subconjunctival area (Fig. **1**).

## DISCUSSION

In this patient, we observed a case of subconjunctival emphysema, decreased air bubble in the AC and low IOP in DSAEK eye. Fortunately, however, the donor tissue adhered to the host corneal stroma without any dislocation and it has been uneventful at following post-operative visits.

At present, there are discussions regarding the endothelial exposure to the air. In fact, some doctors aspirate the air from the anterior chamber just a few minutes after the DSAEK surgery, while most others leave the air as long as possible. In addition, some report DSAEK with C_3_F_8_ or SF_6_ in place of filtered air [8]. In this air exposure issue, therefore, we have to handle the dilemma of how to balance between the corneal endothelial damages and donor adherence. At this point, it is hard to answer this question clearly, because each case has differences in the donor and host corneal conditions, surgeons’ preference, and patient efforts to keep lying down.

Subconjunctival emphysema in this case seemed to be from the temporal-inferior paracentesis incision. We experienced this for the first time, though the DSAEK surgery for this patient was the same as usual. Of course, we may have experienced the air leakage many times without knowing it, because air could leak to the atmosphere, not the subconjunctival space. In that point, emphysema and low IOP worked as good indicators that we could not control the incision and air reduction was faster than humour production.

An unstable incision over the subconjunctival sclera part might be beneficial to the corneal endothelium, because air leakage reduces the exposure time, and because the subconjunctival space is much cleaner than outer conjunctiva or cornea.

However, there are three major problems with an unstable incision. The first is that we cannot predict or control the rate of air leakage. The second is that the probability of endophthalmitis might be still higher, either due to a weak wound, or to fluid flow across the cornea and into the anterior chamber resulting from low IOP, or a combination of both. The third is that we could have higher rate of DSAEK dislocation due to soft eye [9].

Even if we favor the early air reduction in the anterior chamber we have to control air volume in the anterior chamber. To avoid this complication, we should do careful inspection of each wound and chamber stability prior to concluding surgery. And if needed, a suture should be placed over the paracentesis.

## ABBREVIATIONS

DSAEK: = Descemet's stripping automated endothelial keratoplasty
PKP: = Penetrating keratoplasty
AC: = Anterior chamber
IOP: = Intraocular pressure
